# Resilience of females to acute blood–brain barrier damage and anxiety behavior following mild blast traumatic brain injury

**DOI:** 10.1186/s40478-022-01395-8

**Published:** 2022-06-27

**Authors:** W. Brad Hubbard, Gopal V. Velmurugan, Emily P. Brown, Patrick G. Sullivan

**Affiliations:** 1 Lexington Veterans’ Affairs Healthcare System, Lexington, KY USA; 2 Department of Physiology, University of Kentucky, Lexington, USA; 3 Spinal Cord and Brain Injury Research Center, University of Kentucky, 741 South Limestone St, Lexington, KY 40536 USA; 4 Department of Neuroscience, University of Kentucky, Lexington, USA

## Abstract

Low-level blast exposure can result in neurological impairment for military personnel. Currently, there is a lack of experimental data using sex as a biological variable in neurovascular outcomes following blast exposure. To model mild blast traumatic brain injury (mbTBI), male and female rats were exposed to a single 11 psi static peak overpressure blast wave using the McMillan blast device and cohorts were then euthanized at 6 h, 24 h, 7 d and 14 d post-blast followed by isolation of the amygdala. After mbTBI, animals experience immediate bradycardia, although no changes in oxygen saturation levels or weight loss are observed. Male mbTBI animals displayed significantly higher levels of anxiety-like behavior (open field and elevated plus maze) compared to male sham groups; however, there was no anxiety phenotype in female mbTBI animals. Blast-induced neurovascular damage was explored by measuring expression of tight junction (TJ) proteins (zonula occludens-1 (ZO-1), occludin and claudin-5), glial fibrillary acidic protein (GFAP) and astrocyte end-feet coverage around the blood–brain barrier (BBB). Western blot analysis demonstrates that TJ protein levels were significantly decreased at 6 h and 24 h post-mbTBI in male rats, but not in female rats, compared to sham. Female animals have decreased GFAP at 6 h post-mbTBI while male animals display decreased GFAP expression at 24 h post-mbTBI. By 7 d post-mbTBI, there were no significant differences in TJ or GFAP levels between groups in either sex. At 24 h post-mbTBI, vascular integrity and astrocytic end-feet coverage around the BBB was significantly decreased in males following mbTBI. These results demonstrate that loss of GFAP expression may be due to astrocytic damage at the BBB. Our findings also demonstrate sex differences in acute vascular and behavioral outcomes after single mbTBI. Female animals display a lack of BBB pathology after mbTBI corresponding to improved acute neuropsychological outcomes as compared to male animals.

**Supplementary Information:**

The online version contains supplementary material available at 10.1186/s40478-022-01395-8.

## Introduction

Blast-induced traumatic brain injury (TBI) is common in military settings and biomedical efforts to understand blast-induced TBI in order to treat neurological consequences are on-going [7, 17]. Exposure to even low-level blast (LLB) in military personnel results in adverse symptomatology [5] and a unique form of mild brain injury, herein referred to as mild blast TBI (mbTBI). Neurovascular disruption is a hallmark of clinical presentations of blast TBI (bTBI) [27, 46] as well as that in preclinical blast models [3, 11, 24]. Vascular deficits following blast exposure are well described in the literature and this mechanism is supported by many blast injury mechanisms, including direct cranial transmission of shear stress [10] and hydrodynamic pulse through the circulatory system [6].

In the current study, we focus on vascular and blood–brain barrier (BBB) responses in the amygdala, given its critical role in the organization of fear- and stress-related neuropsychology as well as psychiatric disorders, such as post-traumatic stress disorder (PTSD). bTBI leads to long-term anxiety-like behavior accompanied by glial activation, neuronal loss, and neurodegeneration in the amygdala [42]. Acute loss of tight junction proteins as well as microvascular pathology and degeneration in the brain are described following bTBI [1, 11, 24, 25]. BBB disruption after bTBI is well studied in the past decades [28, 37], however little is known regarding sex differences in the response to bTBI.

There are few preclinical studies of blast exposure that include both male and female animals to detect sex differences following bTBI. McCabe and Tucker [30] nicely detail the few studies that incorporate sex as a biological variable (SABV) in bTBI research. Notably, there are sex differences in the generation of hypothalamic–pituitary–adrenal axis impairment as well as limbic system dysregulation following bTBI [39, 40]. Reports have shown sex differences in the vascular response to impact TBI [22, 33], however no reports have examined the vascular or BBB response in bTBI.

In the current study, we seek to fill this knowledge gap in sex differences in the BBB dysfunction following mbTBI as well as characterize the acute vascular pathology of our single mbTBI model. We hypothesize that sex plays a key factor in acute and on-going vascular outcomes as well as post-mbTBI behavioral outcomes. We also hypothesized that a single mbTBI would result in acute, transient BBB dysfunction. To test these hypotheses, we exposed male and female rats to LLB and examined the temporal progression of BBB disruption and anxiety behavior following mbTBI.

## Methods

### Animals and experimental setup

All of the studies performed were approved by the United States Veterans Affairs Animal Component of Research Protocol (ACORP). Additionally, Lexington VA Vivarium is accredited by the Association for the Assessment and Accreditation for Laboratory Animal Care, International (AAALAC, International) and all experiments were performed with its guidelines. All animal experiments were compliant with ARRIVE guidelines and experiments were carried out in accordance with the National Institutes of Health guide for the care and use of Laboratory animals (NIH Publications No. 8023, revised 1978). Male (~ 260 g average weight) and female (~ 220 g average weight) Sprague Dawley rats (Charles River) were used at 7–8 weeks of age.

Animals were randomly assigned to groups, using random number generators. Researchers were blinded to treatment groups during outcome assessment and data analysis. The animals were housed 2–3 per cage (NexGen™ Rat 1800, Allentown Inc.) and maintained in a 12 h light/12 h dark cycle. Confounding factors were minimized by including various treatment groups in the same cage, ensuring all experimental groups are operated on/analyzed at the same time (especially if the assay required multiple cohorts of animals), and all animals were housed in the same room. All animals were fed a balanced diet ad libitum and water was reverse osmosis generated. Animal numbers are reported within the figure legends. For additional details on common data elements used in this study, see Table 1.

**Table 1:** Common data elements for experimental blast exposure in preclinical studies

*Animal characteristics*	
Species	Rattus norvegicus
Age range	7–8 weeks at mbTBI
Sex	Female and male
Animal vendor	Charles River
Strain	Sprague–Dawley
Weight range	200-240 g female; 240-280 g male
*Animal History*	
Housing	Group housed; 12 h light/dark cycle; food and water ad libitum; AAALAC accredited animal care facility maintained to USDA standards
Anesthetic type	Isoflurane (4% for induction, 2.5% for maintenance)
Anesthetic route	Inhaled
Analgesia type	N/A
Injury severity	Mild (blast)
Number of injury exposures	Single
Euthanasia type	Fatal Plus followed by perfusion and decapitation
*Injury model characteristics*	
Injury model	McMillan Blast Device
Device manufacturer	GLR Enterprises, Nicholasville, KY
Animal stabilization method	Rat is fully inside blast tube and laterally placed with respect to blast source. The thorax and lower body is shielded from direct blast forces while the head is unshielded and constrained inside a mesh netting to prevent head movement
*Blast Elements*	
Blast-induced delivery device	McMillan Blast Device
Pressure wave type	Single blast waves (Friedlander-style over- and under-pressure waves)
Detonation type	Compressed helium driver and Mylar® membrane
Detonation material quantity	10 mil-thick Mylar sheet
Driver gas	Compressed helium
Pressure wave medium	Air
Blast tube cross-sectional area	113 square inches
Blast tube length	22.5 feet
Driven section length	20 feet
Membrane thickness	0.010 inches (0.254 mm/sheet)
Membrane burst method	Active puncture with pneumatic knife
Membrane burst pressure	~ 33 psi
Tube end configuration	Open end
Animal orientation to blast wave	Lateral
Overpressure peak	~ 11 psi
Positive overpressure duration	~ 6.7 ms
Impulse	~ 36 psi*ms
Pressure sensor type	Piezoresistive pressure transducers
Sampling frequency	500 kHz
Body exposure	Head fully exposed; body partially exposed
Protective shielding location	Thorax and lower body partially protected
Protective shielding type	Metal tubing
Primary blast effects	Various
Secondary blast effects	N/A
Tertiary blast effects	N/A
Quaternary blast effects	N/A
Systemic injuries	None
Extracranial injuries	None

### Blast injury device

The McMillan Blast Device (MBD) consists of a cylindrical steel tube, 12-inch internal diameter, separated into a 20-ft. expansion chamber and a 2.5-ft compression chamber. We used compressed helium in the compression chamber and 10-mil-thick (0.254 mm) polyethylene terephthalate (Mylar®) membrane to separate the two chambers (Mylar A; Tekra Corp., New Berlin, WI). Industrial grade compressed helium (American Welding & Gas, Lexington, KY), was filled to approximately 33 psi and then manually ruptured by an 8-point blade affixed to a pneumatic cylinder.

**Fig. 1:**
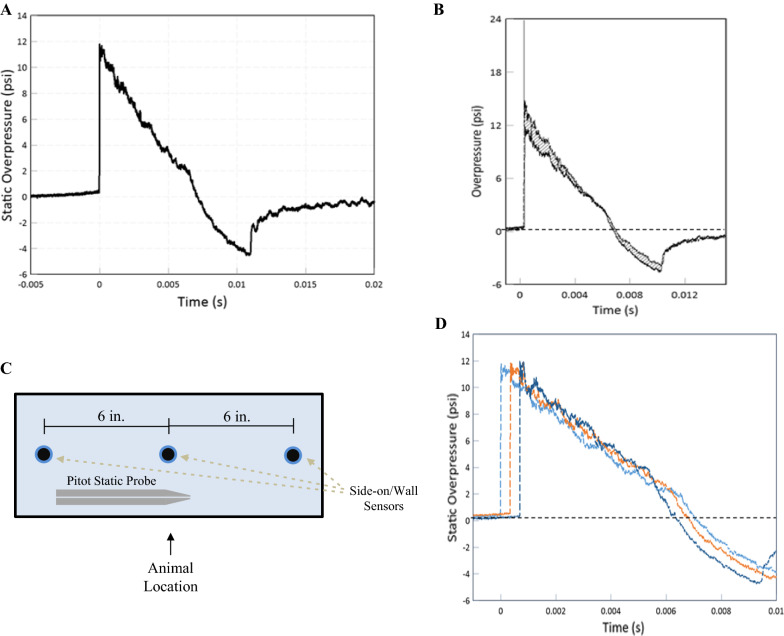
Blast wave characteristics of the McMillan Blast Device. **A** Representative overpressure curve with respect to time measured by a side-on piezoresistive sensor. **B** Overlay of overpressure waves from a face-on sensor of a pitot static probe and a side-on sensor coincident at the same location. Shaded areas represent dynamic overpressure impulses as a relatively minor component of the blast wave. **C** Two-dimensional representation of sensor location along the driven portion of the MBD where the animals are located during blast exposure. **D** Static overpressure traces from the side-on/wall sensors show spatial blast dynamics inside the tube near the animal location

The blast over(under)pressure wave was recorded by a pitot probe (face-on/reflected and side-on) pressure (custom-built; Stumptown Research & Development, LLC, Black Mountain, NC; XTL-190S-100A, Kulite Semiconductor Products, Inc., Leonia, NJ), and piezo-resistive side-on sensors (Model #XTEL-100-190S-100A; Kulite). For the exact sensor locations, see Fig. 1C. Three side-on/wall sensors were positioned equi-distanced (6 inches) around the animal position. Data from each sensor was routed directly to the TMX-18 (AstroNova, Inc., West Warwick, RI). Data were analyzed using AstroView software (AstroNova, Inc.). Data were graphed using FlexPro software.

### Mild blast traumatic brain injury model

Sprague–Dawley rats were transported to the blasting site. The rats were temporarily housed in a climate-controlled room enclosed away from the blast tests and had access to food and water ad libitum during temporary housing. Immediately prior to injury, the rats were anesthetized with isoflurane using SomnoSuite Low-Flow Anesthesia System (Kent Scientific Corporation, Torrington, CT). Rats received 900 mL/min flow at 4.5% isoflurane until fully anesthetized and then placed on a nose cone (400 mL/min at 3.0% isoflurane) for physiologic recordings. Rats were placed into a mesh netting support (Industrial Netting, Minneapolis, MN) and secured into the MBD (two feet from open end of tube) laterally with the left side facing the blast [38]. Once loaded into the MBD, the rat's body was protected from direct forces by a steel tube that surrounded the body but left the head completely exposed to the blast. The mesh netting support restricts head rotation and movement during blast exposure. The rats were subjected to compressed helium-driven blasts at 11 psi peak static overpressure (Fig. 1; blast parameters for each group detailed in Table 2) to model mbTBI. Physiological recordings were taken using SomnoSuite technology (MouseSTAT) five minutes before and five minutes after mbTBI procedure. Rats were monitored and recovered before transportation back to the Lexington VA Vivarium.

**Table 2:** Blast exposure parameters of each experimental group. Data expressed as Mean ± SD

Time point	Group	Peak static overpressure (psi)	Positive overpressure duration (ms)	Positive impulse (psi*ms)	Peak total overpressure (psi)	Peak dynamic overpressure (psi)
6 h	Male	11.88 ± 1.62	6.69 ± 0.27	37.95 ± 2.65	23.93 ± 3.72	12.05 ± 2.14
	Female	11.42 ± 1.44	6.83 ± 0.43	38.42 ± 1.08	22.98 ± 3.43	11.56 ± 2.01
24 h	Male	10.63 ± 2.25	6.98 ± 0.23	36.89 ± 3.02	20.44 ± 5.53	9.81 ± 3.51
	Female	11.63 ± 1.76	6.83 ± 0.36	38.13 ± 2.52	22.66 ± 4.93	11.03 ± 3.27
7d	Male	10.52 ± 1.92	7.03 ± 1.13	34.61 ± 2.60	19.84 ± 5.49	9.32 ± 3.67
	Female	11.82 ± 1.34	6.56 ± 0.29	36.62 ± 3.82	21.92 ± 3.57	10.10 ± 2.44
14d	Male	11.13 ± 1.02	6.67 ± 0.68	36.00 ± 2.35	21.52 ± 2.82	10.39 ± 2.06
	Female	10.74 ± 1.33	6.37 ± 0.28	34.99 ± 2.88	21.52 ± 3.73	10.78 ± 2.47

### Open field test

Open field (OF) testing was performed in the morning hours three days prior to, and two days following low level blast exposure to assess anxiety-like behavior in male and female rats [26]. Rats were placed in a 32″ × 32″ × 12″ dimly lit box for 10 min and their exploration was recorded using Ethovision software. Using software, the box was divided into two zones, with the inner zone being half the size and centered within the outer zone. The software tracked the nose point of the rats and recorded the number of entrances and time spent in each zone. The box was cleaned using 70% EtOH between each test. Exclusion criteria was set based on baseline performance in the acclimation trial (< 10% time in center area).

### Elevated maze plus

Rats were placed in the Elevated Plus Maze (EPM; Med Associates Inc. Fairfax, VT, USA) 7 days following low level blast exposure to assess to assess anxiety in male and female rats [34]. The maze is designed with two open arms, 20″ × 4″, and two closed arms, 20″ × 4″ × 15.94″, with like arms across from each other and an open junction in the middle, 4″ × 4″. The plus maze has no roof, and raised off the ground 29.31″. Rats were placed in the junction between the open and closed arms and allowed to explore for five minutes. The number of entrances into and time spent in either the closed or open arms was recorded using IR beam detection by MedPC software. The lights were slightly dimmed and the box was cleaned between each trial using 70% EtOH.

### Tissue processing

Cohorts of animals were euthanized at 6 h, 24 h, 7 days and 14 days following mbTBI. Rats received intraperitoneal injection of Fatal Plus (Vortech Pharmaceuticals, Dearborn, MI) before transcardial perfusion with cold, sterile saline. After perfusion, rats were decapitated and the brains were then removed from the skull. The left hemisphere of the brains was rapidly dissected to isolate the amygdala and immediately frozen on dry ice. The right hemisphere of the brains was fixed with 4% paraformaldehyde (PFA) for 24 h. Following post-fixation, tissue was placed into 30% sucrose in PBS buffer solution for at least 48 h for cryoprotection. The brain was then flash frozen in − 25 to − 35 °C isopentane before being cut into 40 µm thick coronal sections using a sliding microtome (Microm HM 450, Thermo Fisher). Tissue sections were stored at − 20 °C in cryoprotectant (30% glycerol, 30% ethylene glycol in 1X Tris buffered saline (TBS).

### Western blot

Western blot analysis was performed for tight junction proteins (zonula occludens-1 (ZO-1), Occludin and Claudin-5) and glial fibrillary acidic protein (GFAP) from amygdala brain tissue homogenates. Lysates were made using RIPA buffer (150 mM NaCl, 1% Triton X-100, 0.5% sodium deoxycholate, 0.1% SDS, 50 mM Tris, pH 8.0), centrifuged at 16,100 × G for 30-min and total protein levels were estimated from supernatant using a BCA kit (23225, Thermofisher). Western blot samples were made using XT sample buffer (1610791, Biorad) with DTT and boiled at 95℃ for 10 min. Samples were resolved in duplicate 4–12% BIS–TRIS gels (3,450,125, Biorad) under reducing condition and transferred to PVDF membrane. Probing was done against ZO-1 (1:1000; ZO1-1A12, Thermofisher), Occludin (1:1000; OC-3F10, Thermofisher), Claudin-5 (4C3C2, Thermofisher), GFAP (1:1000; G3893, Sigma) and beta-actin (1:5000; 8H10D10, Cell Signaling). Signals were detected using chemiluminescence substrate (34075, Thermofisher) with anti-mouse IgG-HRP (1:10,000; GENA93, Millipore Sigma), anti-rabbit IgG-HRP (1:20,000; GENA934, Millipore Sigma) or with fluorescence signals using IRDye 68RD goat anti-mouse (1: 10,000; 926–68070, Li-Cor) and IRDye 800 CW goat anti-rabbit (1:10,000; 926–32211, Li-Cor). Protein levels were quantified by densitometric analysis using ImageJ software.

### Immunohistochemistry

PFA fixed, 40 µm thick coronal brain sections were immunostained to examine GFAP expression. Briefly, tissue sections were washed from cryoprotectant and endogenous peroxide activity was blocked with H_2_O_2_ (10% H_2_O_2_ in methanol) for 30 min. Following three time washing with TBS, sections were blocked with blocking buffer contains 5% normal horse serum in TBST (0.1% tween-20 in TBS) and incubated with rabbit anti-GFAP antibody (1: 500; G3893, Sigma) in blocking buffer overnight at 4℃. The next day, sections were washed and incubated with biotin donkey anti-mouse IgG (1: 250; 715–065-15, Jackson Immuno Research) in blocking buffer at room temperature (RT) for 1 h. After rinsing the secondary antibody, sections were incubated with avidin–biotin complex (PK-4000; Vector Laboratories) for 1 h at RT followed by DAB treatment for 2 min as per manufacturers direction. After washing, sections were mounted on glass slides, dried overnight, dehydrated with ethanol and xylene before final permount mounting. Slides were scanned on the Zeiss Axio Scan Z.1 and amygdala regions were traced in HALO for quantification.

### Immunofluorescence

Randomly selected (n = 3–6/group) above mentioned brain sections were double immuno-stained for GFAP (1:250; G9269, Sigma) and SMI-71 (1: 250; 836804, Biolegend) or SMI-71 alone to quantify the astrocyte coverage around the brain vasculature and BBB integrity respectively. Briefly, brain sections were permeabilized in 0.2% Trion X-100 in TBS for 15 mints followed by blocking in blocking buffer (1%BSA+10% normal horse serum+0.1% Triton X-100 in TBS) at RT for 1 h. Then sections were incubated with mixture of rabbit anti-GFAP and mouse anti-SMI-71 or SMI-71 primary antibody in blocking buffer overnight at 4℃. Following day, sections were washed and incubated with mixture of Alexa flour 488 donkey anti-rabbit (1:500; A212206, Invitrogen) and Alexa flour 594 donkey anti-mouse (1: 500; A212203, Invitrogen) or Alexa flour 594 donkey anti-mouse alone as secondary antibody in blocking buffer at room temperature (RT) for 1 h. After rinsing, samples were mounted on glass slides using prolong-glass antifade mount with Nucblue (P36981; Invitrogen). GFAP and SMI-71 double stained slides were scanned and astrocytic end-feet coverage around the blood vessel were analyzed using Nikon confocal microscope (20X; 100X with oil) with NIS-Elements version 5.30.05. Randomly 25 vessels were selected (red channel; SMI 71) in amygdala region from each brain sections (n = 3/group) and measured GFAP mean intensity (green channel) after subtracting the background fluorescence (Additional file 1). SMI-71 stained slides were scanned using BioTek-Cytation-5 with Gen5 Image+3.11 software. Vascular integrity was quantified as vascular density by SMI-71 using ImageJ with vascular density macro.

### Statistical analysis

Power analysis was conducted (using G*Power statistical software; version 3.0.10) for all experimental data and based on previous published literature from our group. Analysis was completed based on the ANOVA statistical tests and output of F score. A priori analysis was performed and effect size was calculated based on expected mean ± SD within each group. Sample size was calculated for behavioral experiments using the following parameters: α = 0.05, 1 − β = 0.8, and standard deviation 20% of mean for experimental groups. Primary outcomes for sample size determination were time in closed arms and tight junction expression. Based on deviation and detectable differences, it was determined that only a subset of the animals was needed to measure IHC and fluorescent histological markers.

Statistical analysis was performed using Graph Pad Prism (GraphPad Software, CA, USA) or JMP 12 (SAS, NC, USA). For all analyses, a significant difference among groups was defined as *p* < 0.05. For each measure, data were measured using interval/ratio scales. The Brown-Forsythe and Bartlett’s tests were performed to ensure homogeneity of variance. Furthermore, the Shapiro–Wilk test was completed to ensure normality. As these criteria were met for all experimental data, parametric statistics were employed for all analyses. Two-way ANOVA test were completed and the Bonferroni post-hoc test was utilized to examine injury effect within sex, where appropriate. Additionally, two-tailed, unpaired t test was also used.

## Results

### mbTBI results in immediate heart rate changes but does not alter oxygen saturation or weight

**Fig. 2:**
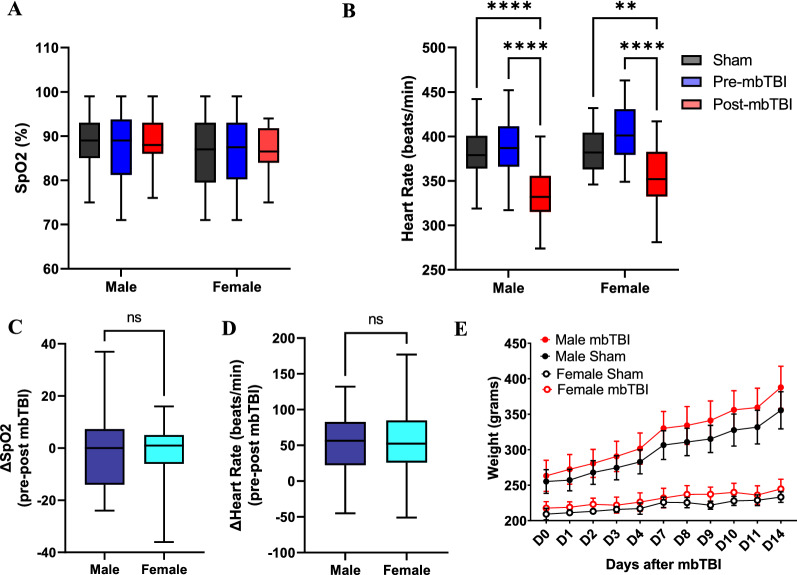
The effect of mbTBI on key physiological parameters in male and female rats. Physiological recordings were taken five minutes before (pre-mbTBI) and five minutes after (post-mbTBI) mbTBI procedure. **A** Box-and-whisker plot represents the peripheral oxyhemoglobin saturation (SpO_2_) and **B** Heart rate (sham, pre-mbTBI and post-mbTBI groups) from male and female rats (n = 28–45/group). **C** Box-and-whisker plot represents the change in SpO_2_ and **D** Heart rate (pre-mbTBI – post-mbTBI) from male and female rats (n = 28–45/group). **E** Daily weight measurement from 0 to 14 d post-mbTBI from male and female rats (n = 6/group). Data are Mean ± Max and Min (Box & Whisker) (**A**, **B**, **C**, **D**) or Mean ± SD (**E**). *P* ≤ 0.002 **, *P* ≤ 0.001 **** by Two-way ANOVA with Tukey’s post-hoc (**A**, **B**, **E**); ns (non-significant) by two-tailed, unpaired t-test (**C**, **D**)

As blast exposure can induce lung damage at higher peak overpressures (> 24 psi) [18, 19, 21, 44], we measured oxygen saturation to ensure our model is of mild blast severity in the absence of overt lung injury. Male and female rats do not have altered oxygen saturation following mbTBI as compared to sham and pre-mbTBI data (Fig. 2A, C). Both male and female rats display bradycardia response immediately following mbTBI compared to sham and pre-mbTBI (Fig. 2B). There were however no differences between sexes (Fig. 2D). There was also no weight change over time in mbTBI animals compared to their respective sham group (Fig. 2E). We demonstrate that mbTBI produces mild physiological effects after injury, including the presence of bradycardia.

### Sex differences in anxiety-related behavior following mbTBI

**Fig. 3:**
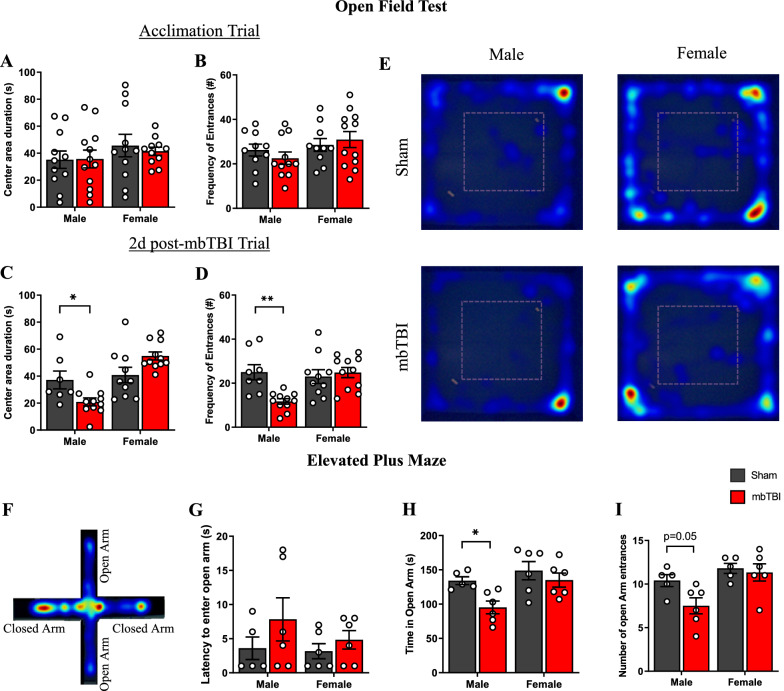
mbTBI alters exploratory and anxiety-related behavior in male rats but not in female rats. Open field test (**A**-**E**) and elevated plus maze (**F**-**I**) from male and female rats. (**A**, **B**) Bar graph represents the total duration (seconds) of time and frequency of entrances to center area of open field during acclimatization at 3 d pre-mbTBI (n = 7–12/group). **C**, **D** Bar graph represents the total duration (seconds) of time and frequency of entrances to center area of open field at 2 d post-mbTBI (n = 11–12/group). **E** Representative heatmap from open field test at 2 d post mbTBI from male and female groups. **F** Representative heatmap from elevated plus maze for a male mbTBI animal. **G** Latency to enter open arm (seconds), **H** total time spent in open arm (seconds) and **I** number of open arm entrances in elevated plus maze at 7 d post-mbTBI (n = 5–6/group). Data represented as Mean ± SEM. *P* ≤ 0.05 *, *P* ≤ 0.01 ** by Two-way ANOVA with Bonferroni post-hoc (**A**, **B**, **C**, **D**, **E**, **G**, **H**, **I**)

Cohorts of rats performed open field exploratory testing before mbTBI and at 2d post-mbTBI. There were no differences in time spent inside the center area or frequency of entrances in center area in the acclimation trial before mbTBI (Fig. 3A, B). However, male mbTBI animals exhibited less center area duration and entrances compared to male sham at 2 d post-mbTBI (Fig. 3C, D), demonstrating anxiety-related behavior of increased thigmotaxia. Female mbTBI however had no significant differences as compared to female sham in open field testing. Representative heat maps of exploration demonstrate the lack of male mbTBI center area exploration (Fig. 3E). Rats from the 14 d cohort underwent elevated plus maze (EPM) testing. Representative heat maps of male mbTBI animals seen in Fig. 3F. Male mbTBI animals displayed less time in the open arms and open arm entrances compared to male sham group (Fig. 3H, I), while there were no differences in EPM parameters between the female groups. There was no difference in latency to enter open arm in both male and female groups (Fig. 3G). These results demonstrate sex-specific presentation of acute anxiety-related behaviors.

### Males, but not females, displayed decreased tight junction protein expression after mbTBI

**Fig. 4:**
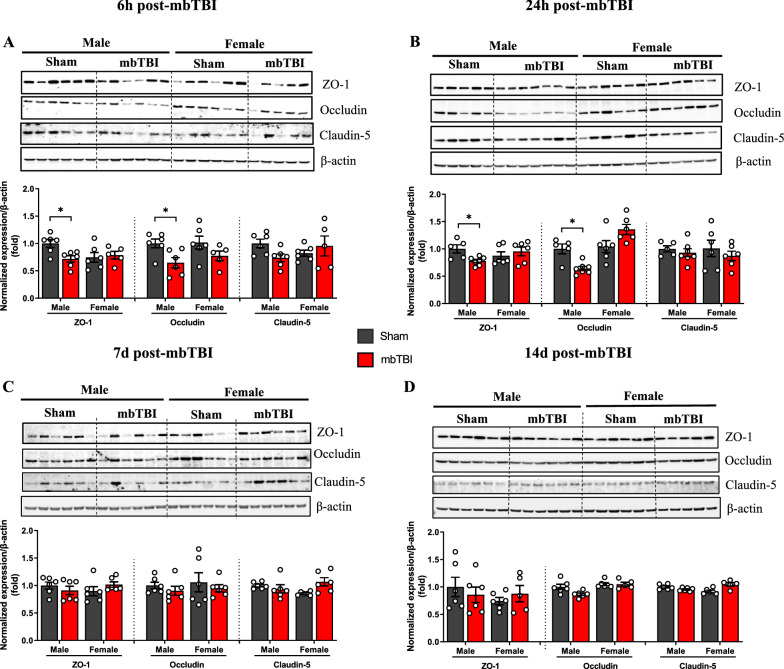
mbTBI decreases acute tight junction protein expression in amygdala of male rats but not in female rats. **A**-**D** Western blot and densitometry quantification of tight junction proteins from amygdala region of male and female rats. Representative western blot and densitometry quantification of ZO-1, Occludin and Claudin-5 from brain amygdala lysates after, **A** 6 h post-mbTBI, **B** 24 h post-mbTBI, **C** 7d post-mbTBI and **D** 14 d post-mbTBI (n = 5–7/group). Data represented as Mean ± SEM. *P* < 0.05 * by Two-way ANOVA with Bonferroni post-hoc (**A**, **B**, **C**, **D)**

Amygdala was dissected at various timepoints after mbTBI to generate a time course response following mbTBI. At 6 h and 24 h post-mbTBI, samples from male mbTBI had significantly less expression of ZO-1 and occludin as compared to male sham (Fig. 4A, B). There is a restoration of ZO-1 back to sham levels at 3 d post-mbTBI (Additional file 2) and occludin back to sham levels at 7 d after mbTBI in male groups (Fig. 4C). Claudin-5 is not significantly changed after mbTBI in male animals. Contrastingly, there were no significant differences in any tight junction protein in the female groups at any time point. These results demonstrate sex-specific acute tight junction protein expression following mbTBI.

### Males display greater acute blood–brain barrier integrity alterations following mbTBI

**Fig. 5:**
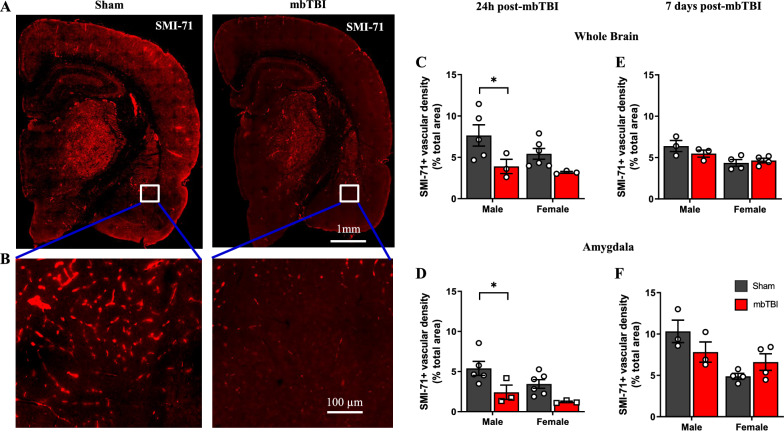
mbTBI affects BBB integrity in male rats but not in female rats. SMI-71 immunofluorescence to assess capillary barrier integrity. **A** Representative SMI-71 immunofluorescence micrograph from Sham and mbTBI rat brain at 24 h post-mbTBI. **B** 20X magnification of vascular integrity in amygdala region. **C**, **D** Vascular density quantification using ImageJ in whole brain section and amygdala region of sham and mbTBI from male and female rats 24 h post-mbTBI (n = 3–6/group). **E**, **F** Vascular density quantification using ImageJ in whole brain section and amygdala region of sham and mbTBI from male and female rats 7 d post-mbTBI (n = 3–4/group) Data represented as Mean ± SEM. *P* ≤ 0.05 * by Two-way ANOVA with Bonferroni post-hoc **C**, **D**, **E**, **F**

To examine BBB integrity, we performed SMI-71 staining, which labels vessels with intact, non-leaky BBB [29, 45]. We found visibly lower amounts of BBB-competent vessels in the brains of male rats following mbTBI as compared to sham (Fig. 5A, B). Male animals display significant decreases in whole brain and amygdala vascular integrity of SMI-71+vessels (Fig. 5C, D) at 24 h following mbTBI as compared to sham. There are lower levels of vascular integrity in females following mbTBI as compared to sham, though these differences are non-significant. By 7 d post-mbTBI, vascular integrity is not significantly different following mbTBI as compared to sham for both sexes (Fig. 5E, F). We show that males experience a greater degree of acute BBB breakdown in the amygdala and whole brain following mbTBI.

### Sex-specific changes in amygdalar GFAP levels following mbTBI

**Fig. 6:**
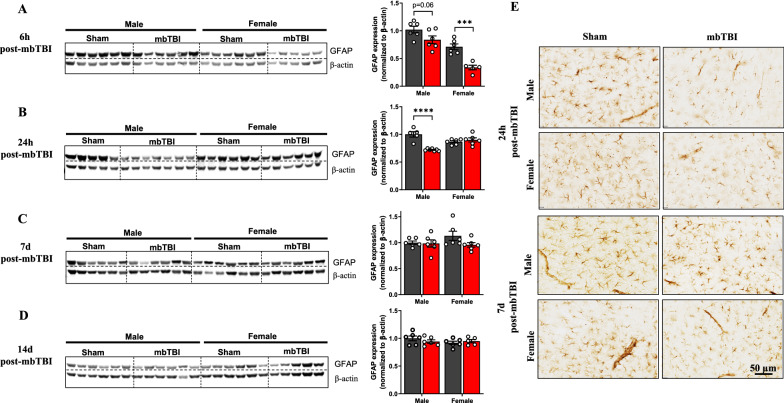
mbTBI decreases astrocyte GFAP expression in early time points after mbTBI. **A**-**D** Western blot analysis of GFAP from amygdala region of male and female rats. Representative western blot and respective densitometry quantification (middle panel) of GFAP from brain amygdala lysates after, **A** 6 h post-mbTBI, **B** 24 h post-mbTBI, **C** 7 d post-mbTBI and **D**14 d post-mbTBI in male and female rats (n = 5–7/group). **E** Representative GFAP immunohistochemistry micrographs from brain amygdala region of male and female rats at 24 h and 7 d post-mbTBI. Data represented as Mean ± SEM. *P* ≤ 0.0001 ****; *P* ≤ 0.001 *** by Two-way ANOVA with Bonferroni post-hoc **A**, **B**, **C**, **D**

Astrocyte responses are critical following TBI and astrocytes have important roles in BBB health. We examined GFAP levels in amygdala following mbTBI. At 6 h post-mbTBI, there are non-significant decreases in GFAP in males and significant decreases in GFAP in females following mbTBI as compared to respective sham groups (Fig. 6A). Contrastingly, at 24 h post-mbTBI, there are significant decreases in GFAP in males and no change in GFAP in females following mbTBI as compared to respective sham groups (Fig. 6B). There is a recovery of GFAP levels after mbTBI in both sexes by 7 d post-mbTBI that is sustained at 14 d post-mbTBI (Fig. 6C, D). Representative micrographs show that change in GFAP expression in the amygdala of male animals at 24 h post-mbTBI and the recovery of GFAP expression by 7 d post-mbTBI (Fig. 6E). We corroborated our western blots results indicating acute GFAP decreases following mbTBI with a subset analysis of GFAP IHC staining (Additional file 3). These results indicate acute changes in amygdalar GFAP expression that are sex-dependent.

### Sex-related alterations in astrocytic coverage of brain vessels following mbTBI

**Fig. 7:**
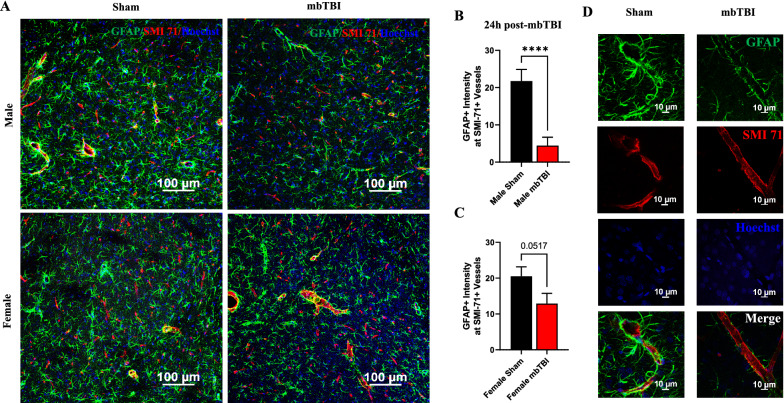
mbTBI decreases astrocyte coverage around brain capillaries after mbTBI. GFAP and SMI-71 double immunostaining was performed to assess astrocyte coverage around the brain capillary. **A**, **B**, **C** Representative confocal micrographs (GFAP-green; SMI-71-red; Hoechst-blue) and respective quantification of GFAP around SMI-71+ brain capillary from amygdala (sham and mbTBI; n = 3 rats/group) of male and female rats (25 vessels from each rat brain amygdala region; n = 75 vessels/group) at 24 h post-mbTBI. **D** Representative high-resolution micrographs (GFAP-green; SMI-71-red; Hoechst-blue) of brain vessel and surrounding astrocytes from male sham and mbTBI rats. Data represented as Mean ± SEM. *P* ≤ 0.0001 **** two-tailed, unpaired t test **B**, **C**

To understand GFAP decreases relative to early pathology following mbTBI, fluorescent co-staining was performed to explain interplay between astrocytes and capillaries. Astrocytic end-feet are tightly intertwined to BBB health. At 24 h post-mbTBI, we observe a decrease in GFAP expression around SMI-71+ vessels in the amygdala of male mbTBI animals (Fig. 7A). Un-merged fluorescent images can be found in Additional file 4. Male mbTBI animals display a greater impairment in astrocytic capillary coverage (Fig. 7B) as compared to female mbTBI animals (Fig. 7C), compared to respective sham groups. High magnification micrographs show the cellular localization in male mbTBI animals at 24 h post-mbTBI (Fig. 7D).

## Discussion

Our preclinical blast model recapitulates appropriate blast physics for modeling military-relevant insults. Static overpressure is the main driving force of injury compared to dynamic forces (Fig. 1B), as seen in other established models [16]. Acute physiology is also pertinent to ensuring clinical relevance and appropriate injury severity. We found no impairments in oxygen saturation or weight change between sham and mbTBI groups. This indicates mild blast severity in our model as opposed to other models that demonstrate severe blast exposure and polytrauma [21]. We did find a decrease in heart rate, or bradycardia, in both sexes following mbTBI. Others report bradycardia following blast exposure, demonstrating an autonomic response and vagal reflex to blast exposure [8, 43]. Additionally, bradycardia has been observed before at low levels of blast exposure [31]. Mild blast severity is further confirmed by the lack of hemorrhage or brain lesions in mbTBI animals compared to sham (Additional file 5).

Anxiety-like behavior resembled that of PTSD is commonly reported following bTBI [20, 35, 41]. The connection between vascular deficits and anxiety-like behavior has been established in the literature [13]. Subacute deficits in elevated maze and open field have already been reported in male rats; these deficits also coincide with transcriptional changes in the amygdala [4]. We found an anxiety-like behavioral response following mbTBI in male animals but not in female animals. This corroborates with Russell, et al. [39], which found that male mice displayed significantly less open arm time and higher closed arm time in EPM following bTBI, whereas there was no significant difference between female groups. The results highlight a potential resilience of female animals to neurological impairments after blast exposure.

Tight junction protein levels are indicative of BBB breakdown following brain injury. Another report show decreases in all tight junction proteins (ZO-1, occludin, and claudin-5) in male animals up to 24 h after bTBI [1], while others [24] find only differences in ZO-1 and occludin following bTBI. In blast-exposed rodents perpendicular to blast source, there was decreased occludin and no changes in claudin-5 at 24 h following single bTBI [16]. Similar to these studies, we found early decreases in ZO-1 and occludin in male animals following bTBI; however, we build upon these findings by showing that TJ protein levels do not change in the amygdala after mbTBI in females. These results highlight an important finding related to sex-divergent BBB response to mbTBI.

SMI-71 has previously been used to assay BBB integrity in bTBI [18, 20]. We found significantly lower levels of SMI-71, which labels vessels with intact, non-leaky BBB [29, 45], in the amygdala of male mbTBI animals compared to sham. SMI-71 levels are reduced in both male and female mbTBI animals, suggesting this may be a more sensitive marker to BBB dysfunction after mbTBI. Again, mbTBI results in greater BBB impairment in male compared to females.

Recent reports demonstrate that military blast exposure can result in lower GFAP in the subpial regions of postmortem brain tissue [2]. We find early decreases in amygdalar levels of GFAP in both male and female animals after mbTBI, however the loss of GFAP is prolonged in male animals. Others have found a lack of GFAP expression after blast exposure, which corresponds with an anxiety-like phenotype [9]. There are no differences in IBA-1 at 6 h post-mbTBI (Additional file 6), which demonstrates a lack of overt microglial loss and suggests that mbTBI preferentially affects astrocytic responses at acute time points. Overall, we find altered time course of GFAP loss after mbTBI that is dependent upon sex.

We then hypothesized that GFAP loss were due to astrocyte damage and disconnection from the microvascular network and BBB. Indeed, we found significant decrease in GFAP intensity around SMI-71 vessels in male animals after mbTBI, demonstrating end-feet reduction and retraction. GFAP disconnections for vascular fractions has been reported at six weeks following repeated mbTBI [12], however we extend this current data to show this at 24 h post-mbTBI. Astrocytic end-feet abnormalities have been described following bTBI [14] and it is shown that GFAP+ astrocytosis occurs in perivascular areas where there is tight junction disruption [28]. A reduction in end-feet coverage has been seen in clinical samples of major depressive disorder [36]. Here, we highlight reduced astrocytic end-feet vessel coverage in perivascular areas around the BBB that is exacerbated in males compared to females following mbTBI.

This study corroborates past studies showing that mild levels of blast exposure can induce acute anxiety presentation accompanied by amygdalar pathology [41]. However, we build upon these findings to show that BBB impairment in the amygdala is worse in male animals compared to female animals after mbTBI. The results of this study agree with the majority of published preclinical TBI research [15], however preclinical research is includes a higher proportion of moderate-severe TBI animal studies in which females experience improved post-injury outcomes. As such, milder forms of TBI are needed to adequately represent the clinical scenario.

There are a variety of variables, including chromosomal, hormonal, and mechanical factors, that can be attributed to the sex-specific differences observed in the current study [30]. The mechanics of blast loading are dependent on body and head size, leading to the possibilities that age-matched male and female animals may generate differential responses due to size differences. Additionally, future study with weight-matched male and female animals could deduce the magnitude of body size on sex differences to blast exposure. It is also highly plausible that there is a hormonal component to this injury response. In an impact acceleration model of TBI, there is an acute increase in BBB permeability and persistent edema in male animals but not in female animals. Ovariectomy in females produced a similar profile to that of males following experimental TBI [33].

There are some limitations to this study. We did not measure hormonal levels in female rats either during blast exposure or at time of euthanasia. Future studies should be designed in incorporate sampling of the estrous cycle at time of injury. We also only examined GFAP+ astrocytic end-feet in vessels that were SMI-71+ ; future studies should examine this in relation to all brain vessels. GFAP only is expressed on a subset of astrocytes and this should be considered with interpretation of this study [23]. Additionally, examining aquaporin-4 would generate results that may be more representative of astrocytic end-feet. Another limitation is that our tight junction results are in brain homogenate and not isolated capillaries; there may be better sensitivity in examining isolated brain capillaries and this will be incorporated in future studies.

This study confirms that there are sex-dependent mechanisms in vascular dysfunction following mbTBI. While it is critical to study both sexes to understand differences in injury progression and treatment, the distinct mechanisms underlying sex-based responses to mbTBI require well-designed experimental design. Of course, there is much to be unraveled and discovered regarding sex-based responses to TBI [32] and there is much to be understood regarding mbTBI and SABV to improve clinical treatment of this injury [7, 30]. Greater understanding of what changes (or does not change) with SABV after blast exposure can reveal appropriate therapeutic targets for bTBI to treat those suffering from bTBI-related neurological impairments. Experimental steps to understand translational challenges, such as differences in sex-based response, are critical to fully understand underlying pathobiology that will lead to improved treatment options.

Our results indicate that male animals have acute decrease in tight junction expression and astrocyte expression in the amygdala that recovers by 7 d post-mbTBI. Astrocytic loss in male animals after mbTBI aligns with a reduction in astrocytic coverage of the blood–brain barrier. Early vascular pathology in males also corresponds to anxiety-related behavioral responses to mbTBI. Further, this study shows that females had greater resiliency to amygdalar blood–brain barrier and vascular dysfunction after mbTBI that corresponds to a lack of subacute anxiety presentation. These results aid our understanding of sex-related differences in the response to mbTBI.

## Supplementary Information

**Additional file 1:** Analysis of astrocyte end feet coverage around brain vasculature.

**Additional file 2:** ZO-1 and occludin levels in the amygdala of male and female rats at 3d post-mbTBI.

**Additional file 3:** Visualization and quantification of GFAP micrographs in the amygdala of male and female rats at 6 h, 24 h, 7 d, and 14 d post-mbTBI.

**Additional file 4:** Unmerged immunofluorescence staining of SMI-71, GFAP, and Hoechst in amygdala at 24h post-mbTBI.

**Additional file 5:** Cresyl violet staining of brain sections demonstrates a lack of morphological change following mbTBI.

**Additional file 6:** There is no change in IBA-1 levels in the amygdala of either male or female rats at 6h post-mbTBI.

## Data Availability

The datasets supporting the conclusions of this article are available at reasonable request.
