# Supplementary material for: Resilience of females to acute blood–brain barrier damage and anxiety behavior following mild blast traumatic brain injury
Source: Acta Neuropathol Commun. 2022 Jun 27;10:93. doi: 10.1186/s40478-022-01395-8 (PMC9235199; doi:10.1186/s40478-022-01395-8)
Supplement: Supplementary file 1 — Additional file 1: Analysis of astrocyte end feet coverage around brain vasculature. [file 40478_2022_1395_MOESM1_ESM.pptx]

## Slide 1
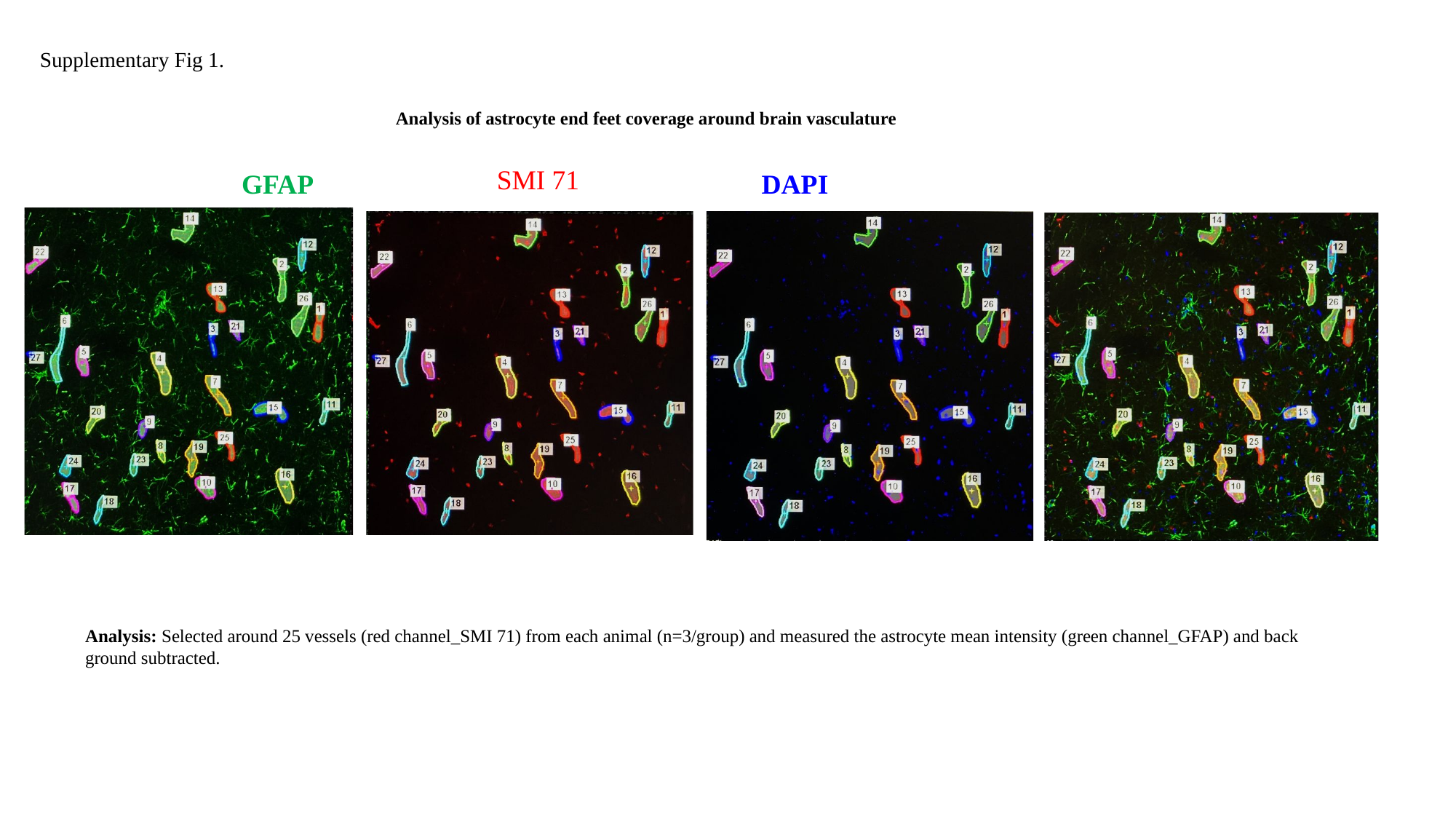

Supplementary Fig 1.
Analysis of astrocyte end feet coverage around brain vasculature
SMI 71
GFAP
DAPI
Analysis: Selected around 25 vessels (red channel_SMI 71) from each animal (n=3/group) and measured the astrocyte mean intensity (green channel_GFAP) and back ground subtracted.
