# Supplementary material for: Resilience of females to acute blood–brain barrier damage and anxiety behavior following mild blast traumatic brain injury
Source: Acta Neuropathol Commun. 2022 Jun 27;10:93. doi: 10.1186/s40478-022-01395-8 (PMC9235199; doi:10.1186/s40478-022-01395-8)
Supplement: Supplementary file 2 — Additional file 2: ZO-1 and occludin levels in the amygdala of male and female rats at 3d post-mbTBI. [file 40478_2022_1395_MOESM2_ESM.pptx]

## Slide 1
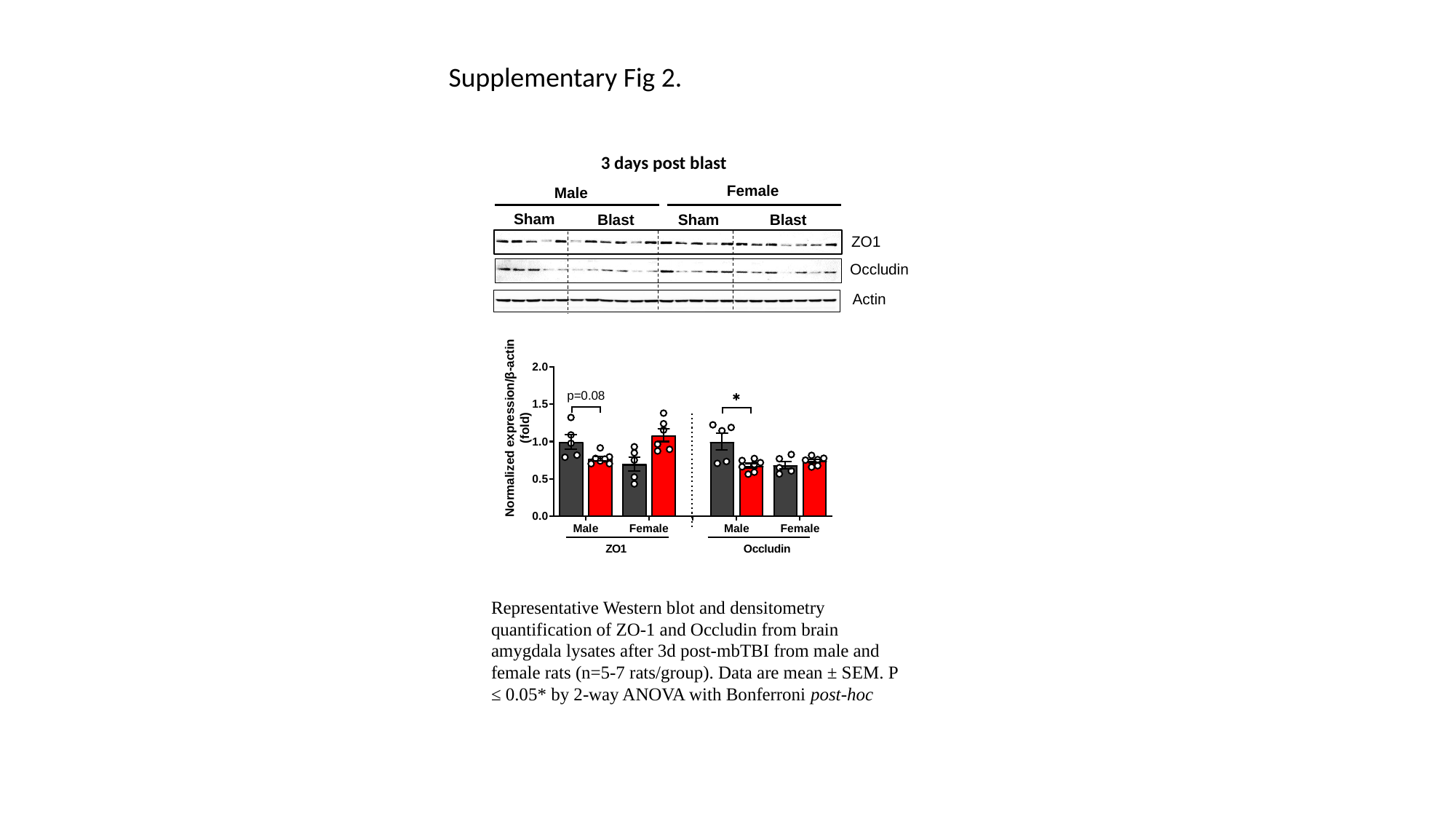

Supplementary Fig 2.
3 days post blast
Female
Male
Sham
Sham
Blast
Blast
ZO1
Occludin
Actin
Representative Western blot and densitometry quantification of ZO-1 and Occludin from brain amygdala lysates after 3d post-mbTBI from male and female rats (n=5-7 rats/group). Data are mean ± SEM. P ≤ 0.05* by 2-way ANOVA with Bonferroni post-hoc
