# Supplementary material for: Resilience of females to acute blood–brain barrier damage and anxiety behavior following mild blast traumatic brain injury
Source: Acta Neuropathol Commun. 2022 Jun 27;10:93. doi: 10.1186/s40478-022-01395-8 (PMC9235199; doi:10.1186/s40478-022-01395-8)
Supplement: Supplementary file 4 — Additional file 4: Unmerged immunofluorescence staining of SMI-71, GFAP, and Hoechst in amygdala at 24h post-mbTBI. [file 40478_2022_1395_MOESM4_ESM.pptx]

## Slide 1
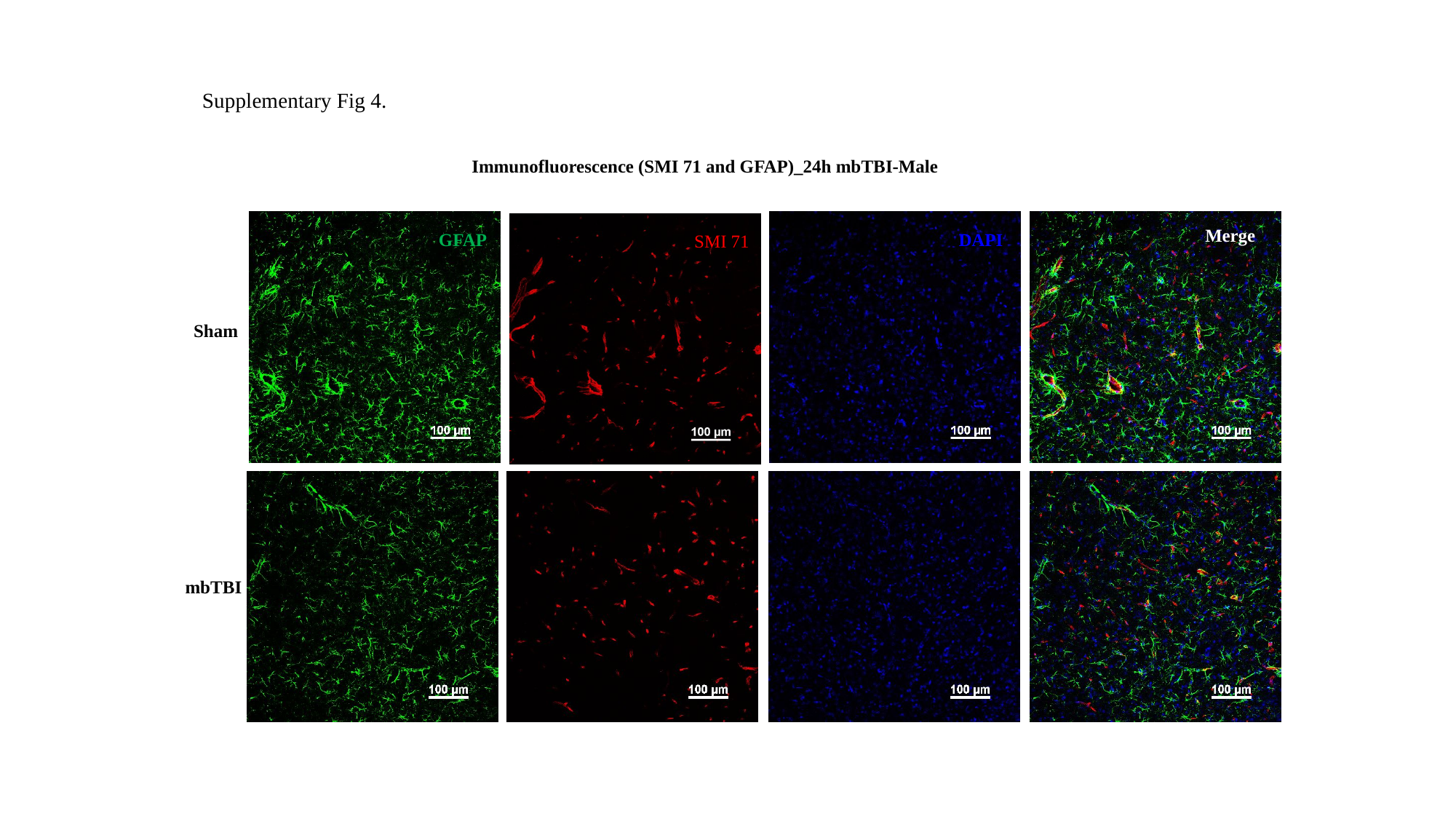

Supplementary Fig 4.
Immunofluorescence (SMI 71 and GFAP)_24h mbTBI-Male
Merge
GFAP
DAPI
SMI 71
Sham
mbTBI
