# Supplementary material for: Resilience of females to acute blood–brain barrier damage and anxiety behavior following mild blast traumatic brain injury
Source: Acta Neuropathol Commun. 2022 Jun 27;10:93. doi: 10.1186/s40478-022-01395-8 (PMC9235199; doi:10.1186/s40478-022-01395-8)
Supplement: Supplementary file 5 — Additional file 5: Cresyl violet staining of brain sections demonstrates a lack of morphological change following mbTBI. [file 40478_2022_1395_MOESM5_ESM.pptx]

## Slide 1
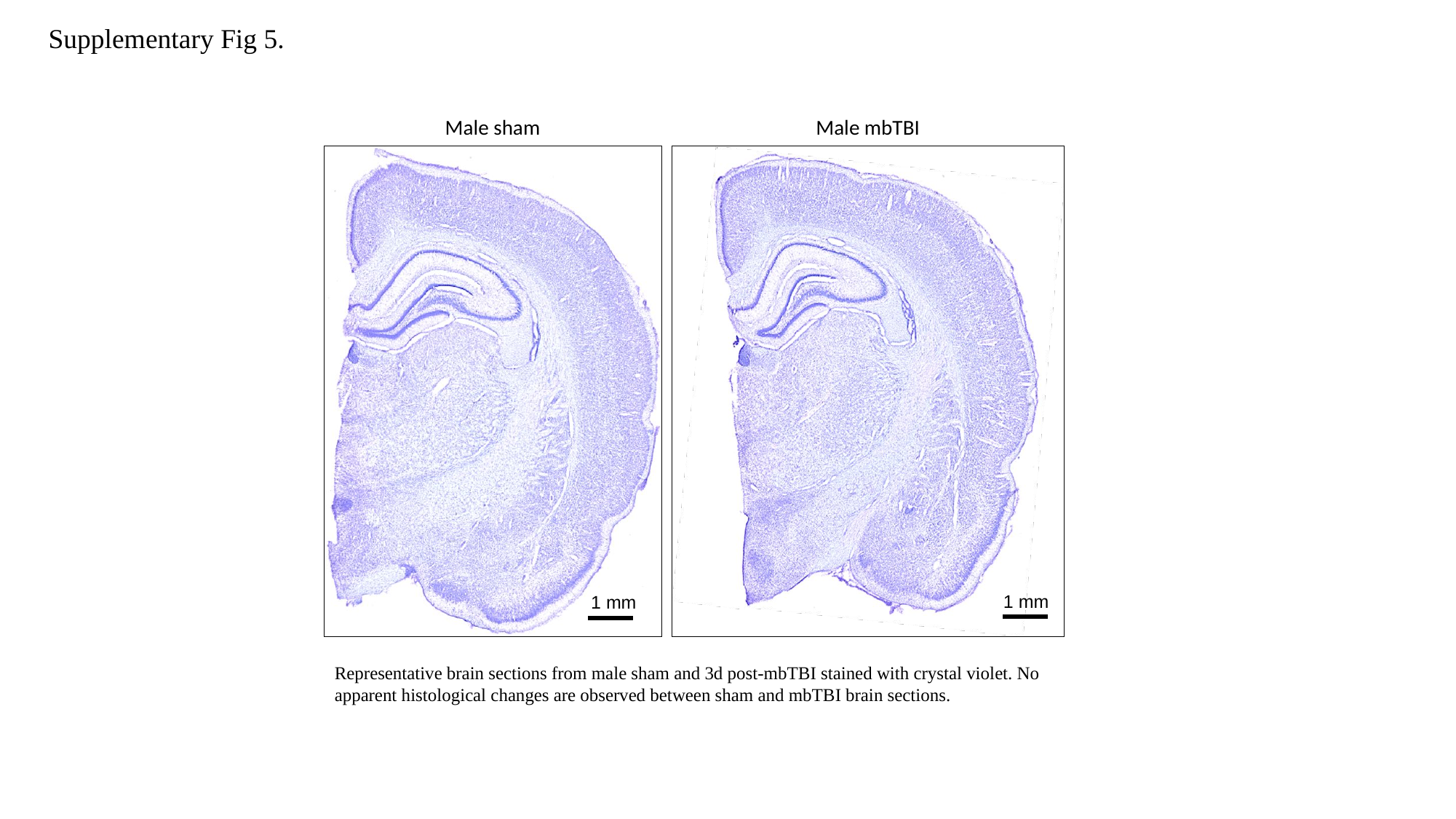

Supplementary Fig 5.
Male sham
Male mbTBI
1 mm
1 mm
Representative brain sections from male sham and 3d post-mbTBI stained with crystal violet. No apparent histological changes are observed between sham and mbTBI brain sections.
