# Supplementary material for: Resilience of females to acute blood–brain barrier damage and anxiety behavior following mild blast traumatic brain injury
Source: Acta Neuropathol Commun. 2022 Jun 27;10:93. doi: 10.1186/s40478-022-01395-8 (PMC9235199; doi:10.1186/s40478-022-01395-8)
Supplement: Supplementary file 6 — Additional file 6: There is no change in IBA-1 levels in the amygdala of either male or female rats at 6h post-mbTBI. [file 40478_2022_1395_MOESM6_ESM.pptx]

## Slide 1
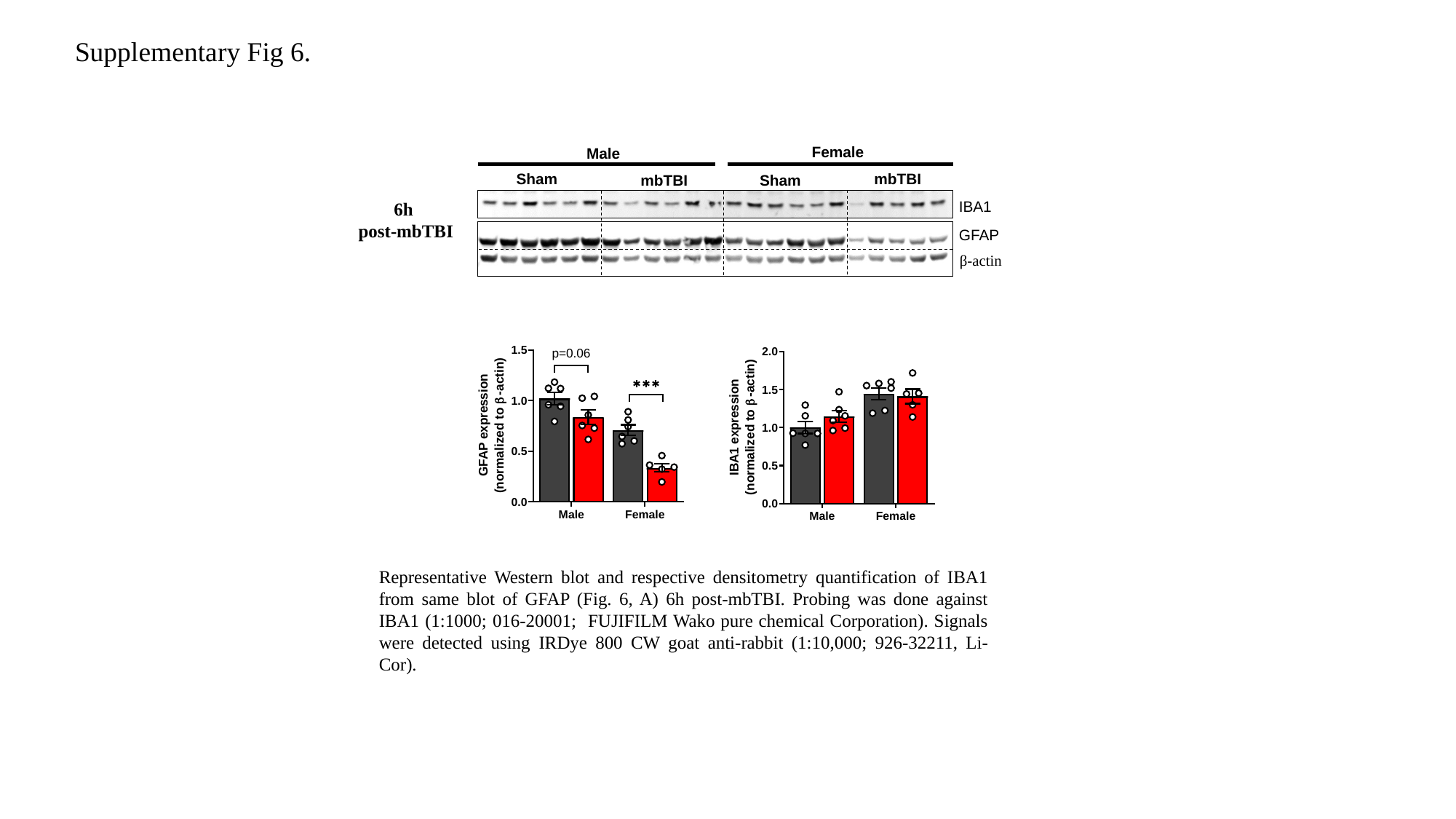

Supplementary Fig 6.
Female
Male
mbTBI
Sham
mbTBI
Sham
IBA1
6h
post-mbTBI
GFAP
β-actin
Representative Western blot and respective densitometry quantification of IBA1 from same blot of GFAP (Fig. 6, A) 6h post-mbTBI. Probing was done against IBA1 (1:1000; 016-20001; FUJIFILM Wako pure chemical Corporation). Signals were detected using IRDye 800 CW goat anti-rabbit (1:10,000; 926-32211, Li-Cor).
